# Scanning Electron Microscopy Study of Retrieved Implants Suggests a Ratcheting Mechanism Behind Medial Migration in Cephalomedullary Nailing of Hip Fractures

**DOI:** 10.5704/MOJ.2003.002

**Published:** 2020-03

**Authors:** GW Law, JSB Koh, AKS Yew, TS Howe

**Affiliations:** Department of Orthopaedic Surgery, Singapore General Hospital, Singapore

**Keywords:** medial migration, pertrochanteric hip fractures, intramedullary nail, scanning electron microscopy, light microscopy

## Abstract

**Introduction:**

Medial migration is the paradoxical migration of the femoral neck element (FNE) superomedially against gravity with respect to the intramedullary component of the cephalomedullary device, increasingly seen in the management of pertrochanteric hip fractures with the intramedullary nail. We postulate that the peculiar anti-gravity movement of the FNE in the medial migration phenomenon stems from a ratcheting mechanism at the intramedullary nail-FNE interface, which should inadvertently produce unique wear patterns on the FNE that can be seen with high-powered microscopy. By examining the wear patterns on retrieved implants from patients with medial migration, our study aims to draw clinical correlations to the ratcheting mechanism hypothesis.

**Material and Methods:**

Four FNEs were retrieved from revision surgeries of four patients with prior intramedullary nail fixation of their pertrochanteric hip fractures complicated by femoral head perforation. The FNEs were divided into two groups based on whether or not there was radiographic evidence of medial migration prior to the revisions. Wear patterns on the FNEs were then assessed using both scanning electron microscopy and light microscopy.

**Results:**

Repetitive, linearly-arranged, regularly-spaced, unique transverse scratch marks were found only in the group with medial migration, corresponding to the specific segment of the FNE that passed through the intramedullary component of the PFNA during medial migration. These scratch marks were absent in the group without medial migration.

**Conclusion:**

Our findings are in support of a ratcheting mechanism behind the medial migration phenomenon with repetitive toggling at the intramedullary nail-FNE interface and progressive propagation of the FNE against gravity.

## Introduction

In recent years, load sharing devices such as fixation with intramedullary nails have gained popularity in the management of pertrochanteric hip fractures^1^. These cephalomedulary devices offer advantages such as a more efficient load transfer with the shorter lever arm, significantly less soft tissue disruption, shorter operative time, and have been shown to have superior outcomes when compared to the traditional extramedullary sliding screw devices particularly in unstable, multifragmentary fractures (AO type A2/A3)^2-7^.

**Fig. 1:**
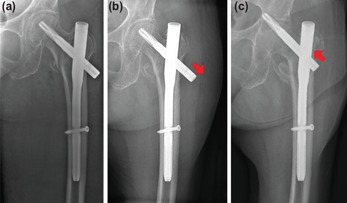
Plain radiographs post fixation of the pertrochanteric left femur fracture with a cephalomedullary device (Synthes proximal femur antirotation nail, PFNA) (a) Immediate postop (b) Subsequent impaction of the fracture with gradual lateral migration of the femoral neck element with respect to the intramedullary nail component (red arrow) (c) Medial migration of the femoral neck element with respect to the intramedullary nail component with perforation of the femoral head and penetration of the acetabulum (red arrow).

Medial migration is a phenomenon seen almost exclusively in the management of pertrochanteric hip fractures with the intramedullary nail (Fig. 1). This is the paradoxical migration of the femoral neck element (FNE) superomedially against gravity with respect to the intramedullary component of the cephalomedullary device, first seen in the description of the Z-effect by Werner-Tutschcku *et al* in their series of 70 proximal femur fractures managed with the Proximal Femoral Nail (PFN)^8^. Medial migration leads to complications with considerable morbidity including femoral head perforation, penetration into the acetabulum, destruction of the hip joint, and in some cases, migration into the pelvic cavity (Table I). This is a poorly understood phenomenon increasingly reported in the literature in the last decade with limited studies investigating the biomechanics of the phenomenon to date (Table I).

**Table I:** Literature on medial migration

Year	Authors	Title	No. of cases	Age / Sex	Morbidity of medial migration	Implant / Construct
2017	Lee JW, Cho HM, Seo JW	Intrapelvic Penetration of Lag Screw in Proximal Femoral Nailing: Case Report	1	72/M	Femoral head perforation with penetration into acetabulum and pelvis	Short Gamma 3 Nail / Lag screw
2016	van Hoef S, Fuchs MCHW, ten Broeke RHM	Late Occurring Medial Migration of Lag Screw in Gamma Nailing	1	81/F	Femoral head perforation with acetabulum penetration	Short Gamma 3 Nail / Lag screw
2016	Pinheiro AC, Alpoim B, Félix A, Alves C, Sousa C, Rodrigues	Medial migration of the intramedullary Gamma nail case report	1	92/F	Femoral head perforation with penetration into acetabulum and pelvis	Short Gamma 3 Nail / Lag screw
		The PFNA Proximal Femur	1	67/F	Femoral head perforation	Proximal Femoral Nail
2015	Brunner A, Jo�ckel JA, Babst	Nail in Treatment of Unstable Proximal Femur Fractures Cases of Post-operative Perforation of the Helical Blade into the Hip Joint			with acetabulum penetration	Antirotation / Helical blade
2015	Nagura I, Kanatani T, Inui A, Mifune Y, Kokubu T, Kurosaka	Medial Migration of the Lag Screw in Gamma Nailing System: Case Report	1	92/F	Femoral head perforation with penetration into acetabulum and pelvis	Short Gamma Nail 3 / Lag screw
2014	JJ Liu, LC Shan, BY Deng, JG Wang, Zhu and ZD Cai	Reason and treatment of failure of proximal femoral nail antirotation internal fixation for femoral intertrochanteric fractures of senile patients	3	-	femoral head perforation with penetration into hip joint Femoral head perforation with penetration into acetabulum and pelvis	Proximal Femoral Nail Antirotation / Helical blade
2014	Takasago T, Goto T, Toki S, Hamada D, Yoshioka S, Tonogai I, Tsutsui T, Tamaki Y, Wada K, Sairyo	Intrapelvic Migration of the Lag Screw in Intramedullary Nailing	1	63/F	Femoral head perforation with penetration into acetabulum and pelvis	Short Gamma 3 Nail / Lag screw
2013	Akçay S, Satoğlu IS, Çabuk H,	Pelvic Migration of Lag Screw Following Fixation of an Intertrochanteric Femur Fracture with Proximal Femoral Nail	1	90/M	Femoral head perforation with penetration into acetabulum and pelvis	Proximal Femoral Nail / Lag screw
2011	Takigami I, Ohnishi K, Ito Y, Nagano A, Sumida H, Tanaka K, Shimizu	Acetabular perforation after medial migration of the helical blade through the femoral head after treatment of an unstable trochanteric fracture with proximal femoral nail antirotation (PFNA) case report.	1	79/F	Femoral head perforation with penetration into acetabulum	Proximal Femoral Nail Antirotation / Helical blade
2011	Frank MA, Yoon RS, Yalamanchili P, Choung EW, DO, Liporace FA	Forward Progression of the Helical Blade Into the Pelvis After Repair With the Trochanter Fixation Nail (TFN)	1	87/F	Femoral head perforation with penetration into acetabulum and pelvis	Trochanter Fixation Nail / Helical blade
2010	Li X, Heffernan MJ, Christina Kane C, Leclair	Medial pelvic migration of the lag screw in short gamma nail after hip fracture fixation: case report and review of the literature	1	77/F	Femoral head perforation with penetration into acetabulum and pelvis	Short Gamma 3 Nail / Lag screw
2010	Lucke M, Burghardt RD, Siebenlist S, Ganslmeier A, Sto�ckle	Medial Migration of Lag Screw with Intrapelvic Dislocation in Gamma Nailing—Unique Problem? Report of 2 Cases	2	75/M	Femoral head perforation with penetration into acetabulum and pelvis.	Short Gamma 3 Nail / Lag screw
				68/M	Femoral head perforation with penetration into acetabulum and pelvis.	Short Gamma 3 Nail / Lag screw
2008	Weil Y, Gardner M, Mikhail G, Pierson G, Helfet D, Lorich	Medial migration of intramedullary hip fixation devices: biomechanical analysis	8	-	Distance of medial migration reported (1.9-22.6mm)	Trochanteric Fixation Nail / Helical blade
2006	Tauber M, Resch	Sigmoid perforation after medial migration of lag screw in gamma nailing	1	84/F	Femoral head perforation with penetration into acetabulum and pelvis	Short Gamma Nail / Lag screw
2002	Werner- Tutschku W, Lajtai G, Schmiedhuber G, Lang T, Pirkl C, Orthner E	Intra-und peri-operative Komplikationen bei der Stabilisierung von per- und subtrochantären Femurfrakturen mittels PFN	5	-	-	Proximal Femoral Nail / Lag screw

Weil *et al* proposed that toggling is required for medial migration to occur based on consistent radiological findings of the fracture pattern involving the medial calcar and the greater trochanter seen in their case series of eight pertrochanteric hip fractures where medial migration occurred^9^. They went on to prove their hypothesis with a biomechanical model specifically engineered for toggling to occur and were successful in recreating the medial migration phenomenon in all five different nail designs tested [Synthes TFN, Synthes PFN, Synthes PFNA, Stryker Gamma-3 nail and Smith and Nephew IMHS nail]^9^. To date, there has been no retrieval studies to validate Weil *et al’s* toggling hypothesis.

We postulate that the peculiar anti-gravity movement of the FNE in the medial migration phenomenon stems from a ratcheting mechanism at the intramedullary nail-FNE interface. This allows FNE motion only in one direction while preventing motion in the opposite direction which will inadvertently produce unique wear patterns on the FNE that can be seen with high-powered microscopy as the FNE pivots on the intramedullary nail during toggling.

We aim to further investigate the medial migration phenomenon and the proposed ratcheting mechanism by studying retrieved implants from patients who have undergone revision surgery as a result of the medial migration phenomenon. By examining the wear patterns on the retrieved implants and correlating these patterns with findings from serial radiographs, our study aims to draw clinical correlations to the ratcheting mechanism hypothesis.

## Materials and Methods

Four FNEs (cephalic blades) were retrieved from revision surgeries of four patients with prior fixation of their pertrochanteric hip fractures with the Synthes Proximal Femoral Nail Antirotation (PFNA), complicated by FNE perforation of the femoral head. Radiographic analysis of plain radiographs were performed and the FNEs were divided into two groups based on whether or not there was medial migration prior to the revisions (n=2 per group). Wear patterns on the FNEs were assessed using both scanning electron microscopy (SEM) and light microscopy. Correlations of the FNE wear patterns with findings from their corresponding radiographic analysis was then performed and compared.

The use of the SEM was made in view of its potential for higher magnification and its ability to create an all-in-focus image in the viewing of a 3-dimensional specimen with significant variations in the Z-axis with postprocessing of recorded stacks of through-focus imaging to overcome the depth of field limit^10^.

Extraction of the FNEs during the revision surgeries were performed in accordance to the manufacturer’s recommendations for FNE removal. All FNE were extracted uneventfully using the Synthes PFNA Blade Extraction Set.

Markings were made on the FNEs at regular intervals using a marker pen and numbered to facilitate orientation and localisation of specific scratch marks on the FNE. Standard preparation with application of a 15nm gold coating using a sputter coater to the surface of the FNEs was performed to facilitate visualisation and surface analysis with the SEM [FEI Quanta 650 FEG]. Viewing was performed at 10kV for all magnifications. A montage of 5x magnification was created to facilitate spatial orientation and the FNEs were reviewed systematically in segments at 40x, 80x, 160x and 300x magnification, respectively.

Radiographic analysis was performed using plain radiographs with anterior-posterior (AP) and lateral views. Measurements of the medial migration distance, tip-apex distance (TAD) and identification of the specific segment of the FNE that passed through the intramedullary component of the PFNA during medial migration were made using software tools [CARESTREAM Vue Motion]. Assessment of fracture configuration and position of the tip of FNE within the nine Cleveland zones in the femoral head were also performed.

The light microscope [Olympus SZX12] was used to facilitate pinpointing of the exact location of specific scratch marks on the FNEs to aid correlation with the specific segment of the FNE that passed through the intramedullary component of the PFNA during medial migration. This was performed systematically in segments at 63x and 90x magnification, respectively.

## Results

**Fig. 2:**
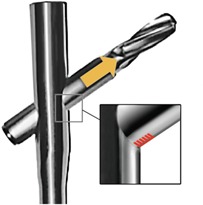
Diagrammatic representation showing repetitive, linearly arranged, regularly spaced transverse scratch marks (red lines) made on the inferior ridge at the intramedullary nail–FNE interface as the FNE migrates medially.

Similar longitudinal scratch marks on both the superior and inferior ridges of the FNE were seen in the retrieval specimens from all four patients. There were however, unique wear patterns present only on the FNEs from the group with medial migration corresponding to the segment of the FNE that has passed through the intramedullary component of the PFNA during medial migration (Fig. 2). These were indentations made by the pivoting action of the FNE on the intramedullary component of the PFNA at the intramedullary nail–FNE interface.

**Fig. 3:**
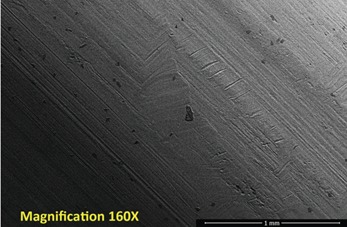
SEM image from inferior ridge of the FNE from Patient 1 with medial migration showing both transverse scratch marks and longitudinal scratch marks. Transverse marks seen only on the apex of the inferior ridge limited to the specific segment of the FNE that traversed through the intramedullary component during medial migration.

**Fig. 4:**
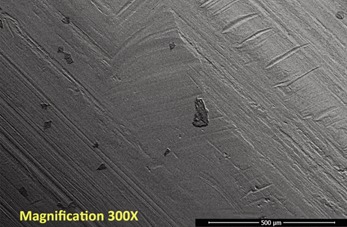
SEM image from inferior ridge of the FNE from Patient 1 with medial migration. On higher powered magnification (300x), more transverse scratch marks can be seen at closer intervals with varying depths.

**Fig. 5:**
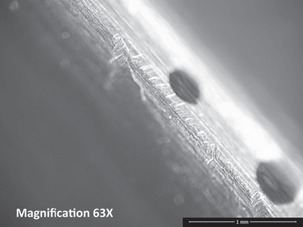
Light microscopy image from inferior ridge of the FNE from Patient 1 with medial migration showing both transverse scratch marks and longitudinal scratch marks (black dots were placed as markers to facilitate identification of the location of the transverse scratch marks on the FNE).

**Fig. 6:**
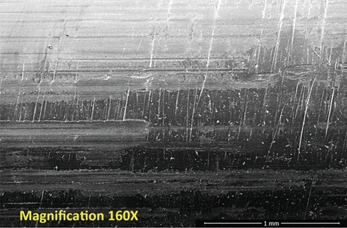
SEM image from inferior ridge of the FNE from Patient 2 with medial migration showing both transverse and longitudinal scratch marks (magnification 160x).

**Fig. 7:**
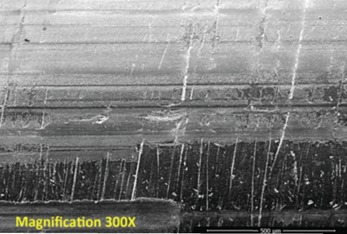
SEM image from inferior ridge of the FNE from Patient 2 with medial migration showing both transverse and longitudinal scratch marks (magnification 300x).

**Fig. 8:**
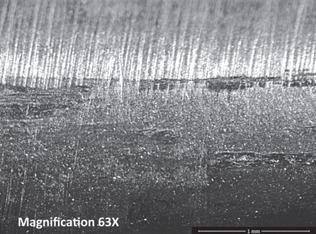
Light microscopy image from inferior ridge of the FNE from Patient 2 with medial migration showing both transverse and longitudinal scratch marks.

Repetitive, linearly-arranged, regularly-spaced transverse scratch marks were seen on the apex of the inferior ridge of the FNE in both patients with medial migration (Fig. 3-8). These are better appreciated on higher powered magnification (300x) with more transverse scratch marks seen at varying depths at closer intervals. These consistent, characteristic scratch marks were found only in the segment of the FNE that traversed through the intramedullary component of the PFNA during medial migration and not elsewhere on the FNE in both patients with medial migration.

The angle that these transverse scratch marks made with respect to the longitudinal axis of the FNE was consistent with the angle that the FNE made with respect to the opening of the intramedullary component of the PFNA at the intramedullary nail–FNE interface at the apex of the inferior ridge (Fig. 7).

These findings were suggestive of (i) repetitive toggling at the intramedullary nail-FNE interface with scratch marks made as a result of a pivoting process at the intramedullary nail–FNE interface when the implant is under load, and (ii) progressive propagation of the FNE superomedially driven by an underlying cyclical process.

**Fig. 9:**
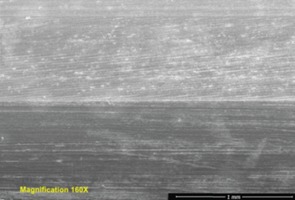
SEM image from inferior ridge of the FNE from patient without medial migration showing only longitudinal scratch marks. No transverse scratch marks were seen.

No transverse scratch marks or scratch patterns unique to a particular part of the FNE was seen on both FNEs in the group without medial migration. Longitudinal scratch marks similar to those found on the FNEs in the patients with medial migration were seen on both the superior and inferior ridges extending through the whole length of the FNE. An example of these longitudinal scratch marks (Fig.9).

Table II, shows a summary of the patients’ demographics, fracture and fixation characteristics, and relevant time points of medial migration and surgery. The mean age was higher in the medial migration group at 85.3 years compared to the group without medial migration at 75.0 years. All patients in our study had BMI less than 20 except for one patient in the group without medial migration who had BMI 30.3. The male to female ratio in both groups were the same. All patients were Chinese.

**Table II:** Summary of patients’ demographics, onset of medial migration, medial migration distance, time points of surgery, fracture pattern and characteristics of fixation.

		Group with medial migration		Group without medial migration	
		Patient 1	Patient 2	Patient 1	Patient 2
Demographics	Gender	Female	Male	Female	Male
	Race	Chinese	Chinese	Chinese	Chinese
	BMI	19.6	14.4	17.8	30.3
Time points	Age at time of index surgery (years)	83.7	86.8	76.1	73.8
	Onset of medial migration post index surgery (months)	2.6	12.3	N/A	N/A
	Medial migration distance (mm)	22.3	12.8	N/A	N/A
	Revision surgery post index surgery (months)	3.6	12.5	4.1	12.8
	Indication for revision surgery	Femoral head perforation with FNE penetration into acetabulum	Femoral head perforation with FNE penetration into hip joint	Femoral head perforation with FNE penetration into acetabulum	Femoral head perforation with FNE penetration into acetabulum
Characteristics of fracture and fixation	Fracture classification (AO/OTA)	31A2.3	31A2.3	31A1.2	31A2.3
	Greater trochanter comminution	Yes	Yes	No	Yes
	Unstable medial calcar	Yes	Yes	No	Yes
	Tip apex distance (mm)	18.9	39.8	15.2	32.9
	Position of tip of FNE within the femoral head (Cleveland zones)	Center-center	Inferior-anterior	Superior-center	Superior- anterior

The medial migration distances seen on radiographs for our patients with medial migration were 22.3mm and 12.8mm seen at 2.6 months and 12.3 months, respectively. The timing of revision surgery were similar in both groups, with one early failure (3-4 months post index surgery) and one late failure (12 months post index surgery).

The indication for revision surgery was FNE perforation of the femoral head in all cases, with penetration into the acetabulum in three of the four cases. The pattern of femoral head perforation however was different between the two groups. In the group without medial migration, superior cutout was seen in both cases with varus collapse of the proximal fracture fragment. In the group with medial migration, cut-out occurred medially in both cases in line with the axis of the femoral neck element, without rotational displacement or varus collapse of the proximal fracture fragment. Unstable fracture configurations were seen in both cases in the group with medial migration and only one case in the group without medial migration (AO/OTA 31A2.3). Comminution at the greater trochanter and an unstable medial calcar pattern were seen in these cases of unstable pertrochanteric fractures.

**Fig. 10:**
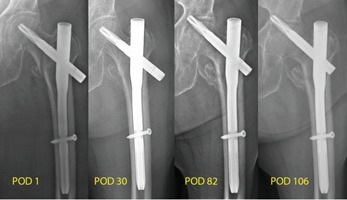
Serial radiographs showing initial FNE lateral migration with fracture impaction and subsequent paradoxical migration of the FNE superomedially against gravity with resultant femoral head perforation and acetabulum penetration in the medial migration group.

Fig. 10 shows the serial post-operative radiographs in the medial migration group with progressive superomedial migration of the FNE leading to fixation failure with femoral head perforation, FNE penetration into the acetabulum and destruction of the hip joint.

**Fig. 11:**
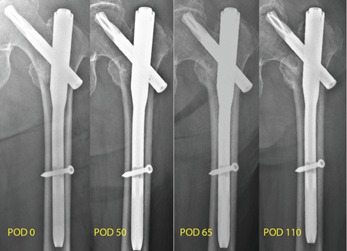
Serial radiographs showing progressive superior cutout of the FNE, varus collapse of the proximal fracture fragment, femoral head perforation and subsequent acetabulum penetration in the group without medial migration.

Fig. 11 shows the serial post-operative radiographs demonstrating femoral head perforation, FNE penetration into the acetabulum and varus collapse of the proximal fracture fragment in the group without medial migration.

TAD were 18.9mm and 39.8mm in the group with medial migration, 15.2mm and 32.9mm in the group without medial migration. The position of the FNE tip within the femoral head were center-center and inferior-anterior in the group with medial migration, superior-center and superior-anterior in the group with medial migration.

## Discussion

Weil *et al* proposed that toggling is required for medial migration of the femoral neck element in the cephalomedullary device to occur based on the consistent fracture pattern involving the medial calcar and the greater trochanter seen in their case series of eight pertrochanteric hip fractures where medial migration occurred^9^. In our group with medial migration, consistent findings of an unstable pertrochanteric fracture configuration (AO/OTA 31A2.3) were found in all patients with deficits seen in both the medial calcar and the greater trochanter similar to Weil *et al’s* case series of patients.

Weil *et al’s* toggling theory was supported by their biomechanical study where they were successful in recreating the medial migration phenomenon in all five different nail designs tested [Synthes TFN, Synthes PFN, Synthes PFNA, Stryker Gamma-3 nail and Smith and Nephew IMHS nail] with a biomechanical model specifically engineered for toggling to occur. No medial migration was seen when toggling was intentionally restricted in all of the cephalomedullary nail designs with a single femoral neck element tested. In our study with the Synthes PFNA which has a single femoral neck element, we found repetitive, linearly-arranged, regularly-spaced transverse scratch marks only on the FNEs from the group with medial migration, corresponding to the segment of the FNE that has passed through the intramedullary component of the PFNA during medial migration. These characteristic wear patterns were indentations made by the pivoting action of the FNE on the intramedullary component of the PFNA at the intramedullary nail-FNE interface, suggestive of repetitive FNE toggling and progressive migration of the FNE driven by an underlying cyclical process, in support of Weil *et al’s* toggling theory. The longitudinal scratch marks found common to all retrieval FNEs may have been made during the insertion of the FNE with the hammer or during the removal of the FNE with the slotted hammer.

Medial migration was also observed in dual lag screw intramedullary nail systems, in the Z-effect phenomenon^8^. Interestingly, preventing nail toggle did not prevent medial migration of the distal FNE when two femoral neck implants [Synthes PFN] were used in Weil *et al’s* study, suggesting that the mechanism of migration in two-screw devices may be different^9^. Migration was prevented only with clamping of the nail and removal of the superior neck element^9^.

Cephalomedullary nail fixation devices have a significantly lower primary cut-out rate compared to extramedullary devices^11-17^. This is supported by Sommer *et al’s* biomechanical study showing higher cut-out resistance in the intramedullary constructs versus extramedullary constructs when implants with similar FNE designs were tested [FNE screw designs: Stryker Gamma nail versus Synthes DHS; FNE blade designs: Synthes TFN versus Synthes DHS]^18^.

Despite being more resistant to cut-out, femoral head cut-out remains as the most common complication of cephalomedullary nail fixation in the management of pertrochanteric hip fractures of which the majority is believed to be the result of biomechanical failure^11, 12^. Medial migration occurs in a subset of these cut-outs and has an increasing number of cases reported in the literature in the last decade. With wear patterns demonstrated on retrieved implants clearly different from other cut-outs suggesting different underlying mechanisms leading to failure, further studies may be beneficial in investigating whether the subset of cut-outs due to medial migration are different from the regular femoral head cut-outs well described in the literature.

Interestingly, Sommer *et al’s* study also showed that the FNE blade designs [Synthes TFN, Synthes DHS] outperformed the FNE screw designs [Stryker Gamma nail, Synthes DHS] in terms of overall cut-out resistance, suggesting that the FNE blade is a superior design compared to the FNE screw irrespective of whether of whether it is an intramedullary or extramedullary construct^18^. In Nikoloski *et al’s* study of 6 cut-outs in 97 patients managed with the Synthes PFNA which has a FNE with a helical blade design, a bimodal distribution of TAD leading to failure was seen^19^. All cut-outs occurred had TAD either less than 20mm or more than 30mm. No cut-outs occurred with TAD between 20-30mm. Nikoloski *et al* proposed that the helical blade behaves differently to a screw, and that placement too close to the subchondral bone may lead to penetration through the head. Our study findings were similar to Nikoloski’s study with all cut-outs occurring either less than 20mm or more than 30mm in both groups.

**Fig. 12:**
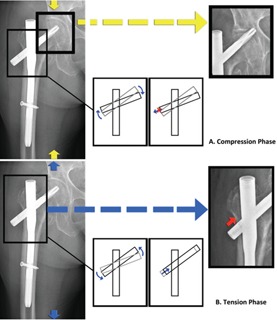
Diagrammatic representation of postulated mechanism behind the medial migration phenomenon with repeated loading-unloading at the hip joint.

Based on the wear patterns seen in our study unique to the group with medial migration, we postulate that medial migration requires two criteria to occur: (i) toggling, and (ii) propagation of the femoral neck element medially with respect to the proximal fracture fragment. This is a progressive process where perforation of the femoral head and acetabulum occurs during the compression phase (e.g. single leg stance), and propagation of the femoral neck element medially occurs during the tension phase (e.g. when the lower limb is lifted off the ground). Fig. 12 shows a diagrammatic representation of the postulated mechanism. The clockwise moment of the femoral neck element during the compression phase prevents lateral migration of the femoral element allowing perforation of the femoral head to occur while the anticlockwise moment of the femoral element during the tension phase results in propagation of the femoral neck element medially with respect to the intramedullary component. This leads to FNE motion only in one direction while preventing motion in the opposite direction similar to a ratcheting mechanism and would account for the anti-gravity movement of the FNE seen in the medial migration phenomenon. Given that toggling of the FNE is a bi-directional movement, potential driving factors behind this progressive process of medial migration could include activities that involve repeated cycles of loading-unloading at the hip joint such as during gait, transfers or stance changes. This is predisposed in the setting of unstable pertrochanteric fracture configurations with risk factors including (i) comminution at the greater trochanter resulting in the lack of a proximal lateral buttress for the intramedullary nail, (ii) insufficiencies at the medial calcar either from an unstable fracture pattern or from poor reduction and (iii) fracture non-union.

The risk of cut-out has been associated with FNE tip positioning within the femoral head^20-26^. Cleveland *et al* divided the femoral head into nine zones with good reliability in the reporting of FNE tip positioning^26, 27^. Caruso *et al* in their retrospective analysis of 571 patients for cut-out risk predictors in cephalomedullary nailing of pertrochanteric fractures showed the highest rates of cut-out occurring with superior FNE tip placement within the femoral head^26^. Interestingly, this pattern of FNE tip placement resulting in cut-outs was seen only in the group without medial migration in our study. In the group with medial migration, cut-outs occurred despite FNE tip placement in low risk positions. This is especially significant as it includes the center-center position, commonly thought to convey the highest resistance to cut-outs^22, 24, 26^. Larger studies will be useful in the assessment of whether the cutout risk with respect to FNE tip positioning in medial migration follows the regular pattern seen in cut-outs given the unique underlying pathophysiology in medial migration. Our study is the first retrieval study in the literature investigating the medial migration phenomenon. With high-powered magnification and the scanning electron microscopy’s ability to create an all-in-focus image in the analysis of the 3-dimensional FNE specimen, we were able to perform a detailed and comprehensive examination of the wear patterns on the FNE specimens to verify the toggling mechanism hypothesis. The consistent, unique wear patterns found on the retrieved FNE specimens exclusive to the medial migration phenomenon serves as strong evidence in support of Weil *et al’s* toggling mechanism hypothesis, in line with the radiological and biomechanical findings from their study.

One limitation in our study is our small sample size. Despite the increasing number of cases reported in the last decade, medial migration remains poorly recognised to date and retrieval specimens are difficult to acquire. Although our study findings are convincing of repetitive toggling occurring at the intramedullary nail-FNE interface with progressive migration of the FNE in the medial migration phenomenon, evidenced by unique, consistent wear patterns present only in the specific segment of the FNE that passed through the intramedullary component of the PFNA during medial migration, retrieval studies with larger sample sizes will be useful in confirming our findings. With toggling being a bi-directional process predisposed by an unstable pertrochanteric fracture configuration, and progressive FNE migration likely driven by an underlying cyclical process, biomechanical studies with bi-directional cyclic loading at the hip joint may be useful in investigating the role of loading-unloading at the hip in the medial migration phenomenon, particularly in unstable pertrochanteric fractures.

## Conclusion

The wear patterns found on the FNE with medial migration are in support of repetitive FNE toggling and progressive migration of the FNE, driven by an underlying cyclical process. Coupled with radiological findings of a one-direction motion of the FNE superomedially against gravity, our study findings are suggestive of a ratcheting mechanism exclusive to the medial migration phenomenon.
